# Thoracotomy vs. Thoracoscopy for Esophageal Atresia with Tracheoesophageal Fistula Repair: Is There a Difference in Quality of Life?

**DOI:** 10.3390/children11111340

**Published:** 2024-10-31

**Authors:** Donatella Di Fabrizio, Francesca Mastroberti, Alba Cruccetti, Edoardo Bindi, Giovanni Cobellis

**Affiliations:** 1 Pediatric Surgery Unit, Salesi Children’s Hospital, 60123 Ancona, Italy; francesca.mastroberti@ospedaliriuniti.marche.it (F.M.); alba.cruccetti@ospedaliriuniti.marche.it (A.C.); edoardo.bindi@ospedaliriuniti.marche.it (E.B.); giovanni.cobellis@ospedaliriuniti.marche.it (G.C.); 2 Department of Specialized Clinical and Odontostomatological Sciences, University Politecnica of Marche, 60121 Ancona, Italy

**Keywords:** esophageal atresia, thoracotomy, thoracoscopy, quality of life, children

## Abstract

Aim: Esophageal atresia (EA) is the most common congenital esophageal malformation. Thoracoscopic repair is gaining popularity, but controversy persists regarding the ideal surgical approach for this challenging anomaly. This study’s aim is to analyze outcomes in terms of quality of life (QoL) of children undergoing thoracotomy and thoracoscopy for type III EA repair. Methods: Perinatal characteristics, malformations, surgical procedures, complications, gastrointestinal, and respiratory current symptoms were collected. QoL was investigated through Esophageal-Atresia-QoL (EAQoL) and Pediatric-QoL (PedsQL) 4.0 standardized and validated questionnaires. Statistical Mann–Whitney test with significance level *p* < 0.05 was carried out. Main results: A total of 32 patients undergoing type III EA primary repair answered the questionnaire, including 17 thoracotomy and 15 thoracoscopy. Median birth weight was not significantly different between two groups (median [2.385; 2.475], *p* = 0.3529) while gestational age showed statistically difference (median [36; 38], *p* = 0.0063). Thirty-five associated malformations (15 thoracotomy, 20 thoracoscopy) in 23 patients were observed. Postoperative complications occurred in nine patients in the thoracotomy group (one recurrent fistula, two leakages, six stenosis) and in six patients in the thoracoscopic group (two recurrent fistula, four stenosis). Analyzing EAQoL, we found statistically significant difference in physical health between the two groups (median [83; 96], *p* = 0.0119), while there was no difference in social relations, eating, and total score (median [100; 100], *p* = 0.3473) (median [91; 97], *p* = 0.5749) (median [91; 96], *p* = 0.1710). Investigating PedsQL, psychosocial health score (median [88; 98], *p* = 0.0069), and total score (median [91;99], *p* = 0.0043) were statically significant different between the groups, whereas there was no difference for physical health score (median [91; 100], *p* = 0.0706). Conclusions: Thoracoscopic EA repair is safe and feasible, allowing patient to have a better QoL in terms of physical and psychosocial health score.

## 1. Introduction

Esophageal atresia (EA) is the most prevalent congenital anomaly affecting the esophagus, occurring in approximately 1 in 3000 to 4000 live births [1,2]. This condition results from an embryonic defect characterized by a disruption in the normal development of the esophagus [3], often involving multigenic factors and epigenetic modifications of genes [4,5]. As a consequence, various types of esophageal discontinuities can manifest, with or without the presence of a tracheoesophageal fistula (TEF), as classified by the Gross Classification system [6]. It is not uncommon for EA patients to present with additional associated anomalies or defects affecting other organ systems, significantly impacting their management and prognosis [5,7].

Advancements in intensive care, anaesthesia, ventilatory and nutritional support, and surgical techniques have all played pivotal roles in achieving a significant outcome enhancement for individuals with EA over the last two decades, with survival rates now surpassing 90% [1,8,9,10].

Ongoing advancements in surgical technology and techniques, coupled with a multidisciplinary approach to care, are continuously refining our understanding and management of complex EA cases, further improving outcomes for affected individuals. Specifically, the introduction of thoracoscopy has revolutionized the approach to EA repair by offering a minimally invasive alternative to traditional thoracotomy. This technique presents several advantages, including reduced postoperative pain, minimal scarring, and a lower risk of skeletal deformities [11]. However, it is important to note that thoracoscopy has not entirely replaced thoracotomy. While thoracoscopy offers numerous benefits, it remains a challenging technique that requires extensive training and expertise. Consequently, both approaches continue to be utilized based on individual patient characteristics and the preferences and skills of the surgical team.

As mortality rates for EA have decreased, there has been a growing emphasis on assessing functional outcomes and quality of life (QoL) in affected individuals [12,13]. Assessing QoL provides insights into the holistic well-being and functional status of individuals beyond mere survival outcomes. Various aspects of life can be significantly impacted by the long-term complications associated with EA, spanning from gastrointestinal and respiratory function to nutrition, social activities, and relationships, ultimately influencing overall QoL [13].

To date, there are multiple studies evaluating QoL in EA patients, focusing on understanding how this congenital condition affects individuals over the long term [14,15], but none of them compares thoracoscopy and thoracotomy in terms of quality of life.

The aim of this study was to comprehensively analyze the long-term complications and outcomes in terms of QoL of children undergoing thoracotomy and thoracoscopy for type III EA repair.

## 2. Methods

A retrospective analysis involving children who underwent thoracotomy or thoracoscopy for repair of type III EA was carried out. This study included all children diagnosed with type III EA and treated by the same surgeon (GC) at the Pediatric Surgery Unit of Salesi Children’s Hospital between 2014 and 2022, with a follow-up period of at least 1 year. Thoracoscopic esophageal atresia (EA) repair was introduced at our institution in 2016 following specialized training, and all patients treated with this method by one surgeon since then were included in the study.

Participants were categorized into two groups: those who underwent thoracotomy for repair of type III EA and those who underwent thoracoscopic repair for the same condition.

The medical records were carefully examined for personal and perinatal information, associated malformations, details of surgical interventions and complications, gastrointestinal and respiratory current symptoms, and actual anthropometric data. Additionally, Quality of Life (QoL) was investigated through Esophageal Atresia QoL (EA QoL) [14] and Pediatric QoL (Peds QL) 4.0 [16] standardized and validated questionnaires.

Prior to the start of the survey, all parents provided consent for the retrospective analysis of anonymized data for the purpose of clinical research.

### 2.1. Perinatal Data

Perinatal details such as sex, gestational age, and birth weight were recorded for children with type III EA according to Gross Classification [6].

Associated anomalies were divided into cardiovascular, gastrointestinal, anorectal, urogenital, limb, skeletal, respiratory, central nervous system, and other malformations (eye, ear, VACTERL association, CHARGE syndrome, chromosome trisomy).

### 2.2. Surgical Information

The thoracotomy approach was performed via a right 5th intercostal space. Through the intrapleural space, the TEF was identified, ligated with sutures, and divided. The upper esophageal stump was mobilized, and an end-to-end esophagoesophageal anastomosis was performed using interrupted sutures, with a trans-anastomotic orogastric tube positioned before to complete the anastomosis. A paranastomotic chest drain was placed and the thoracotomy closed in layers. In the thoracoscopic approach, the patient was positioned semi-prone, and a 5 mm trocar was inserted below the right scapula tip, followed by a 3 mm and a 5 mm trocar under direct vision. The mediastinal pleura was incised, and the TEF was closed with two clips. The upper esophageal stump was isolated, and an end-to-end esophagoesophageal anastomosis was performed with a trans-anastomotic orogastric tube in place. A paranastomotic chest drain was inserted through the posterior trocar site, and the port sites closed in layers.

Primary surgery details included the type of procedure performed, such as primary repair, prior gastrostomy with delayed anastomosis, staged repair, and other procedures for associated malformations. Secondary surgery such as revisional surgery for recurrence of TEF, dilations for stenosis treatment, and laparoscopic Nissen fundoplication surgery for gastroesophageal reflux were recorded.

We also analyzed post-surgical complications including recurrence of tracheoesophageal fistula, anastomotic stenosis or leakage, and diagnosis of gastroesophageal reflux.

### 2.3. Follow-Up and Clinical Outcome

Follow-ups were scheduled every 3 months during the first year of life, every 6 months during the subsequent two years, and then annually until adolescence.

Digestive symptoms, feeding difficulties, and respiratory disorders were assessed since the beginning of the study. Digestive symptoms included dysphagia, thoracic burn, painful swallowing, regurgitation, and vomiting problems. Feeding difficulties comprised avoiding certain foods, eating small portions, adjusting food consistency, needing more than 30 min per meal, increasing fluid intake during meals, nutrition through gastrostomy, or infusion pump. Respiratory symptoms included cough, wheezing at physical activity, dyspnea at physical activity, airway infection, chest tightness, and asthma.

### 2.4. Anthropometric Data

Information on the children’s current weight (in kilograms) and height (in centimeters) were collected. Growth percentile for weight and height was calculated on current age using CDC growth charts [17].

### 2.5. Quality of Life Questionnaire

Since 2023, the quality of life in all patients has been systematically evaluated using the standardized and validated questionnaires Esophageal Atresia Quality of Life (EA QoL) and the Pediatric Quality of Life Inventory (Peds QoL) 4.0.

The specific scale EA QoL by Dellenmark-Blom et al. (2017) [14], adapted for different age groups, included:

EA-QoL version for children aged 2–7 years, completed by parents, encompassing 17 items including three categories: eating, physical health, and social relations.

EA-QoL version for children aged 8–12 years, provided by parents, comprising 24 items covering four categories: eating, social relationships, body perception, and physical health.

Each item was scored on a 5-point Likert scale, with higher scores indicating better quality of life, and final scores were transformed to a 0 to 100 scale.

The generic scale Peds QoL 4.0 [16] adapted for different age groups included:

Peds QoL version for infants aged 1–12 months, filled out by parents, including 36 items comprising physical functioning and symptoms, emotional, social, and cognitive functioning.

Peds QoL version for infants aged 13–24 months, completed by parents, containing 45 items involving physical functioning and symptoms, emotional, social, and cognitive functioning.

Peds QoL version for toddles aged 2–4 years, answered by parents, comprising 21 items covering physical functioning, emotional, social, and day care functioning.

Peds QoL version for young children aged 5–7 years, provided by parents, covering 23 items concerning physical functioning, emotional, social, and school functioning.

Peds QoL version for children aged 8–12 years, answered by parents, including 23 items about physical functioning, emotional, social, and day care functioning.

Each item was assessed reporting the frequency of each problem on a 5-point Likert scale, with higher scores indicating better quality of life, and final scores were transformed to a 0 to 100 scale.

### 2.6. Statistical Analysis

The demographic characteristics of patients with EA were summarized using descriptive statistics. For normally distributed quantitative data, we reported the mean ± standard deviation (SD). Independent sample *t*-tests were employed to compare differences between groups. For non-normally distributed quantitative data, we provided medians with interquartile ranges. Mann–Whitney U tests were utilized to compare differences between groups. Categorical data were compared between groups using chi-squared (χ2) tests or Fisher’s exact tests. A significance level of *p* < 0.05 was used for statistical significance. All statistical analyses were conducted using Graphpad Prism 10.3 software (Version 10.3).

## 3. Results

A total of 34 children, born with type III EA, were treated between 2014 and 2022 at the Pediatric Surgery Unit of Salesi Children’s Hospital. Thirty-two patients answered the questionnaire, with a response rate of 94.1%, including 17 thoracotomy and 15 thoracoscopy.

### 3.1. Perinatal Data

The gender was not statistically different between the two groups, including 9 (53%) males in the thoracotomy group (TCg) and 9 (60%) in the thoracoscopy group (TSg) (Table 1).

Median birth weight was not significantly different between two groups (median [2.385; 2.475] kg, *p* = 0.3529), while gestational age showed statistical difference (median [36; 38] weeks, *p* = 0.0063), displaying that premature babies mostly underwent thoracotomy rather than thoracoscopy.

In the TCg, 13 (87%) patients had 15 associated malformations, comprising interatrial or interventricular defect with cardiac shunt (7), Fallot’s tetralogy (1), aortic arc anomalies (2), duodenal atresia (1), anorectal malformation (1), first metacarpal agenesis (1), and tracheomalacia (2).

In the TSg, there were 20 associated malformations in 10 (67%) patients, including interatrial or interventricular defect with cardiac shunt (6), hypoplastic left heart (1), pulmonary artery stenosis (1), cardiac isthmic stenosis (1), Hirschsprung disease (1), anorectal malformation (1), hydronephrosis (1), double renal ectopic kidney (1), cystic igroma (1), hemi vertebrae (2), subependymal cyst (1), vascular tracheal compression (1), preauricular appendix (1), and George’s Syndrome (1). The number of associated malformations did not statistically differ between the two groups.

### 3.2. Surgical Information

In the TCg, 16 (94%) children underwent primary repair for EA, while 1 (6%) child required gastrostomy placement and delayed repair. Additionally, 2 (12%) children underwent other surgeries for associated malformations: one underwent a posterior sagittal anorectoplasty according to Pena for anorectal malformation, while the other underwent a duodenoduodeno-anastomosis according to Kimura for duodenal atresia.

In the TSg, 15 (100%) children underwent primary repair for EA. Among them, 3 (20%) children required other surgeries for associated malformations: one underwent colostomy followed by laparoscopic-assisted posterior sagittal anorectoplasty for anorectal malformation, another underwent tracheostomy followed by cardiac surgery for vascular tracheal compression causing respiratory failure, and the third underwent a laparoscopic-assisted endorectal pull-through for Hirschsprung disease.

No intraoperative complications occurred in both groups.

Postoperative complications occurred in 9 (53%) patients in the TCg, including 1 (6%) case of recurrent TEF requiring revisional surgery and later on a dilatation program, 2 (12%) cases of leakages managed conservatively, and 6 (35%) cases of stenosis necessitating an endoscopic dilatation program.

In the TSg, 6 (40%) children experienced postoperative complications, comprising 2 (13%) cases of minimal recurrent TEF managed conservatively and 4 (27%) cases of stenosis requiring an endoscopic dilatation program. Additionally, one patient underwent thoracoscopic revision due to suspicion of recurrent TEF, which yielded negative results.

Furthermore, gastroesophageal reflux disease (GERD) required surgical correction with minimally invasive Nissen’s fundoplication in 3 (18%) children from TCg and 1 (7%) child from TSg, performed between the ages of 1 and 7. The median number of dilatations required for stenosis was 5 (range 1–5) in the TCg and 2 (range 1–5) in the TSg. The number of concomitant surgeries for associated malformations and the number of postoperative complications did not statistically differ between the two groups.

### 3.3. Follow-Up and Clinical Outcome

The mean follow-up was 7.3 ± 2.5 years for the TCg and 4.9 ± 1.7 years for the TSg.

**Figure 1:**
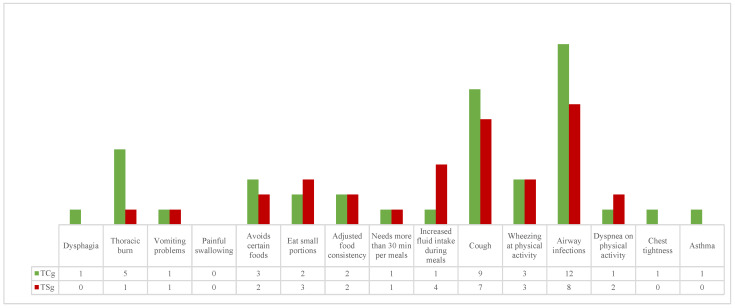
Digestive symptoms, feeding difficulties, and respiratory symptoms occurring in the two groups in the past year.

During the last year of follow-up, 13 (76%) children in the TCg had 43 symptoms: 5 patients had digestive symptoms (1 dysphagia, 5 thoracic burn, and 1 vomiting problems), 4 patients had feeding difficulties (3 avoiding certain foods, 2 eating small portions, 2 adjusting food consistency, 1 needing more than 30 min per meal, and 1 increasing fluid intake during meals), and 12 children had respiratory symptoms (9 cough, 3 wheezing at physical activity, 1 dyspnea at physical activity, 12 airway infection, 1 chest tightness, and 1 asthma) (Figure 1).

In the TSg, 13 (87%) children had 34 symptoms in the last year: 2 patients had digestive symptoms (1 thoracic burn and 1 vomiting problems), 6 patients had feeding difficulties (2 avoiding certain foods, 3 eating small portions, 2 adjusting food consistency, 1 needing more than 30 min per meal, and 4 increasing fluid intake during meals), and 12 children had respiratory symptoms (7 cough, 3 wheezing at physical activity, 2 dyspnea at physical activity, and 8 airway infection) (Figure 1).

Digestive and respiratory symptoms, as well as feeding difficulties, did not show statistically significant differences between the two groups (respectively, *p* = 0.4025, *p* = 0.6911, *p* = 0.4501).

### 3.4. Anthropometric Data

The patient’s mean age was 6.7 ± 2.4 years in the TCg and 4.5 ± 1.6 years in the TSg.

In the TCg, the average weight of the population was 23.4 ± 8.5 Kg, located at the 19th (IQR 9–19) percentile, while the average height was 118.7 ± 22.2 cm, found at the 33rd (IQR 28–70) percentile.

In the TSg, 16.1 ± 3.2 Kg was the average weight of the population, located at the 30th (IQR 14–55) percentile, while 104.4 ± 12.0 cm was the average height, placed at the 38th (IQR 12–82) percentile.

### 3.5. Quality of Life Questionnaire

Both the EA QoL and the Peds QoL questionnaires were completed by parents. In the TCg, 12 parents (71%) were mothers, with a mean age of 43.6 ± 7.9 years. In the TSg, 13 parents (87%) were mothers, with a mean age of 37.7 ± 6.5 years.

EA QoL in the TCg showed a median of 91 (IQR 88–97) for eating, 83 (IQR 83–95) for physical health, and 100 (IQR 100) for social relations in children between 2 and 7 years, while there was a median of 94 (IQR 84–97) for eating, 80 (IQR 75–82) for physical health, 100 (IQR 83–100) for social relations, and 100 (IQR 97.5–100) for body perception in children between 8 and 9 years.

The total score for EA QoL in children undergoing thoracotomy for EA repair was 91 (IQR 89–93).

EA QoL in the TSg revealed a median of 97 (IQR 88–100) for eating, 96 (IQR 88–100) for physical health, and 100 (IQR 100) for social relations in children between 2 and 7 years.

The total score for EA QoL in children undergoing thoracoscopic EA repair was 96 (IQR 91–99).

**Figure 2:**
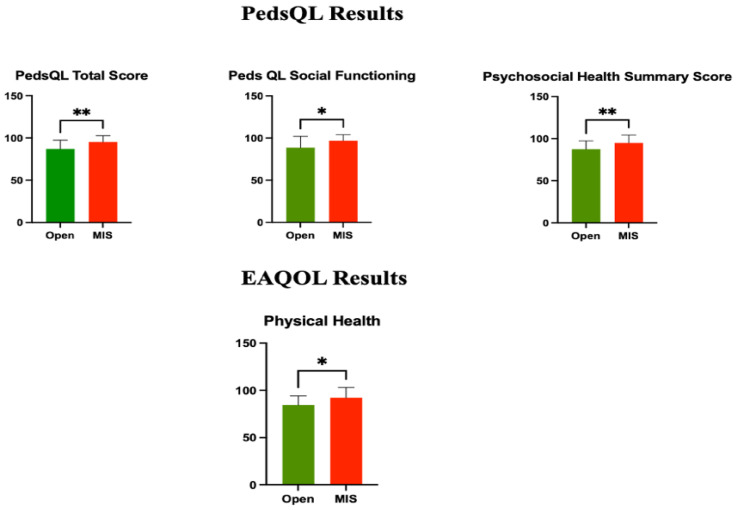
Quality of life scores. Examining PedsQL, total score (*p* = 0.0043), social functioning (*p* = 0.0474), and psychosocial health score (*p* = 0.0069) were statistically significantly different; while analyzing EAQoL, we found a statistically significant difference in physical health (*p* = 0.0119). * *p* ≤ 0.05, ** *p* ≤ 0.01.

Analyzing EA QoL, there was no statistical difference in social relations (*p* = 0.3473), eating (*p* = 0.5749), and total score (*p* = 0.1710), but we found a statistically significant difference in physical health between the two groups (*p* = 0.0119), demonstrating that thoracoscopic repair has a significant impact on physical health (Figure 2).

In the TCg, the Peds QoL assessment revealed the following medians and interquartile ranges (IQRs): 91 (IQR 75–100) for physical functioning, 95 (IQR 80–100) for emotional functioning, 90 (IQR 80–100) for social functioning, and 90 (IQR 73–100) for school functioning. The physical health summary score was 91 (IQR 75–100), the psychosocial health summary score was 88 (IQR 85–93), and the total score was 91 (IQR 82–94).

In the TSg, the Peds QoL assessment found the following medians and IQRs: 100 (IQR 95–100) for physical functioning, 100 (IQR 90–100) for emotional functioning, 100 (IQR 100) for social functioning, and 100 (IQR 95–100) for school functioning. The physical health summary score was 100 (IQR 96–100), the psychosocial health summary score was 98 (IQR 95–100), and the total score was 99 (IQR 93–100).

There was no statistically significant difference between the two groups in physical functioning (*p* = 0.0706), emotional functioning (*p* = 0.1631), and school functioning (*p* = 0.1310). However, social functioning was found to be significantly different (*p* = 0.0474) between the thoracotomy and thoracoscopy groups, showing that thoracoscopic repair has a significantly positive effect on social functioning. When comparing the Peds QoL summary scores between the two groups, statistically significant differences were observed in the psychosocial health summary score (*p* = 0.0069) and the total score (*p* = 0.0043) in favor of thoracoscopy, while no significant difference was found for the physical health summary score (*p* = 0.0706) (Figure 2).

## 4. Discussion

EA stands as one of the most severe congenital gastrointestinal developmental anomalies, necessitating neonatal surgical intervention. The optimal surgical approach remains a topic of debate and ongoing research within the medical community [18]. To determine the potential superiority of one surgical method over another and to establish consensus among experts, it is crucial to consider postoperative outcome and long-term prognosis.

Postoperative EA patients face potential gastrointestinal and respiratory complications, impacting the long-term prognosis and affecting the children’s lives [19]. Consequently, the health-related quality of life subsequent to surgical correction has emerged as a primary focus for both follow-up and research endeavors.

QoL can be defined as the individuals’ perception of daily life encompassing their physical, mental, and social functioning, influenced by both the disease itself and medical interventions, within the context of the culture and value systems in which they live [20]. The QoL could be assessed using generic and condition-specific tools: generic instruments offer broader applicability, facilitating comparisons between patients and healthy individuals, like the Ped QoL 4.0 [17], while condition-specific tools specifically analyze the clinical factors influencing a precise disease, as in our case, the EA QoL [14]. This combination of tools allows for a more comprehensive assessment, capturing both broader aspects of quality of life applicable across populations and more tailored insights specific to the challenges faced by EA patients.

To date, numerous studies in the literature have explored the EA QoL, but this study stands out as the first to compare QoL outcomes between thoracotomy and thoracoscopy, aiming to understand how the selection of surgical approach might impact long-term patient outcomes.

In our research, the two groups were similar in patient number, gender, and birth weight, while the gestational age was significantly different, showing that premature babies are more often treated by thoracotomy EA repair, consistent with previous findings in the literature [21,22].

The most recent meta-analysis between thoracotomy and thoracoscopy, including 1043 patients, showed non-significant differences in the literature in postoperative anastomotic leakage, esophageal stricture, need for endoscopic esophageal dilatation, and mortality between the two techniques [23]. In our report, the rate of revisional surgery for recurrent TEF suspicion did not differ between the groups, while all the other complications were managed conservatively or with an esophageal dilatation program. Strictures were the most common complications, in both groups, with a slight prevalence in the TCg (35% vs. 27%). This finding aligns with the literature, although reported percentages often hover around 40% [24]. The frequency of endoscopic dilatations needed for stenosis and the requirement for Nissen fundoplication for GERD were more common in the TCg, although statistical significance was not observed.

In the past year, a significant portion of children in both groups exhibited symptoms, with slightly higher occurrences noted in TCg compared to TSg. The number of children with respiratory symptoms was equal in both groups, prevailing over digestive symptoms in both cases, although with minor distinctions in the types of respiratory issues. The observed pulmonary health challenges across all subgroups might stem from disrupted respiratory tract development and maturation, potentially influenced by factors like tracheomalacia, airway infections, and asthma, [25,26] and it is probably not related with the type of surgical technique adopted. Gastrointestinal symptoms, slightly higher in TCg, were mainly dysphagia, gastroesophageal reflux, and feeding difficulties. The abnormal motility of the esophagus continues to be the primary pathophysiological factor contributing to digestive morbidity in children with EA; indeed, it is crucial not only for the movement of food from the mouth to the stomach, but also plays a pivotal role in protecting the esophagus against gastric reflux. The etiology of esophageal dysmotility could be mainly attributed to an abnormal development of esophageal smooth muscle, intrinsic innervation, and issues with the vagus nerve, but it is worth noting that surgical interventions with fibrotic scarring and postoperative complications may exacerbate esophageal dysmotility [26,27,28]. In our series, it appears that the surgical technique does not significantly influence the occurrence of esophageal dysmotility; however, further prospective studies involving larger patient cohorts are necessary to thoroughly investigate this aspect.

Despite the complexity of EA, the recent literature shows a quite good quality of life of EA patients, often comparable to that of their peers without EA [29,30], probably because children with chronic conditions seems to cope better with everyday health-related problems compared to healthy children [31,32].

It has been shown that generic QoL decreases in cases of prematurity, cardiac malformation, complex esophageal surgery, digestive, and respiratory symptoms [13,29,33,34], while only digestive and respiratory symptoms tend to influence the condition-specific QoL [32,34].

Physical health is one of the most affected domains, likely associated with frequent hospitalizations for complications such as anastomotic stenosis, in addition to other medical issues like heart defects, tracheomalacia, and recurrent respiratory symptoms [29]. Analyzing the specific EA QoL scores, we found that the physical health score was positively and significatively influenced by the minimally invasive approach in children, with a better score in the EA patient undergoing thoracoscopic EA repair. Even if there was not a statistically significant difference, in the TSg, eating and total specific score were higher than the TCg.

Moreover, a reduced score in social functioning is observed when the issue of postoperative esophageal motor dysfunction becomes more pronounced and affects the social life [29,32].

Investigating the general PedsQL score, we discovered that the psychosocial health score was clearly statistically different between the groups, underlining that thoracoscopic repair has a positive influence on the psychic and social field of these children.

Furthermore, the total generic QoL score was significantly superior in patients treated by minimally invasive surgery.

The current study presented some limitations. First of all, only a parent-proxy questionnaire was used because the older children able to answer the questionnaire were only in the TCg; therefore, although some studies indicate good agreement between parents and children, there is still the possibility of differences in perception. Secondly, it was a single-center pilot study with a limited sample size and a specific population, thus it is essential to recognize that the findings may not be fully generalizable to other populations or settings.

Moving forward, expanding the sample size and collaborating with other centers can enhance the study’s robustness and validate the findings across diverse populations.

## 5. Conclusions

Based on the findings of the current study, it seems that thoracoscopic surgery offers EA patients improved QoL in both physical and psychosocial health domains. Nevertheless, confirmation of these results would benefit from multicenter studies involving larger sample sizes.

## Figures and Tables

**Table 1:** Demographics of population. The gestational age was significantly different between the two groups (bold).

Variables	Thoracotomy Group (n = 17)		Thoracoscopy Group (n = 15)		*p* Value
	Absolute Number of Patients (%)	Median (IQR)	Absolute Number of Patients (%)	Median (IQR)	
Demographics					
Sex, males	9 (53%)		9 (60%)		
Gestational Age (weeks)		36		38	**0.0063**
Birth weight (kg)		2.385		2.475	0.3529
			
Associated anomalies	13 (87%)		10 (67%)		0.5382
			
Surgery					
Primary repair	16 (94%)		15 (100%)		0.9999
Prior gastrostomy and late repair	1 (6%)		0 (0%)		0.9999
Other procedures	2 (12%)		3 (20%)		0.6454
			
Complication occurrance	9 (53%)		6 (40%)		0.5023
Recurrent TEF	1 (6%)		2 (13%)		0.5887
Leakage	2 (12%)		0 (0%)		0.4859
Stenosis	6 (35%)		4 (27%)		0.7120
GERD requiring surgery	3 (18%)		1 (7%)		0.6029
			
Auxological parameters					
Weight		19° (9–19)		30° (14–55)	0.8888
Height		33° (28–70)		38° (12–82)	0.6209

## Data Availability

Data available on request from the corresponding author.

## Abbreviations

EA	Esophageal atresia
TEF	Tracheoesophageal fistula
QoL	Quality of life
EAQoL	Esophageal atresia QoL
PedsQL	Pediatric QoL 4.0
TCg	Thoracotomy group
TSg	Thoracoscopic group
GERD	Gastroesophageal reflux disease
SD	Standard deviation
IQR	Interquartile range
